# Full-Genome Sequence of an Enterovirus 71 Strain Isolated from a Throat Swab from a Child with Severe Hand-Foot-and-Mouth Disease in Changzhou, China, in 2017

**DOI:** 10.1128/genomeA.01439-17

**Published:** 2018-01-25

**Authors:** Qiong Li, Feng-Ming Wang, Xu-Jian Mao, Ping Yao, Xia Jiang, Jing-Yi Jiang, Cong Chen, Fei-Fei Hu, Jun-Hong Li

**Affiliations:** a Inspection Department, Changzhou Center for Disease Prevention and Control, Changzhou, Jiangsu, People’s Republic of China; b Institute of Acute Infectious Disease Prevention and Control, Changzhou Center for Disease Prevention and Control, Changzhou, Jiangsu, People’s Republic of China

## Abstract

The full-length genome sequence of a human enterovirus 71 (EV71) strain (EV71/CZTN01/CHN/2017) was isolated from a throat swab from a child in Changzhou, China, in 2017. According to the phylogenetic analyses, the full-genome sequence in this study belongs to sub-subgenotype C4a.

## GENOME ANNOUNCEMENT

Hand-foot-and-mouth disease (HFMD) is a common epidemic disease of global concern that usually affects children under the age of 5 years. Clinical symptoms are typically mild and self-limiting, but rarely, patients may also develop neurological complications, such as encephalomyelitis, aseptic meningitis, and acute flaccid paralysis, or even death within a few days (1). In the past three decades, multiple severe HFMD outbreaks have been documented throughout the world, especially in the Asia-Pacific region, in countries such as Taiwan, China, Japan, Malaysia, Singapore, and even Australia (2, 3).

Enterovirus 71 (EV71) is a group of naked positive single-stranded RNA viruses. The genome of EV71 is about 7.4 kb, comprising a 5′ untranslated region (UTR), P1 structural polypeptide, P2 and P3 nonstructural polypeptides, and a 3′ UTR containing a long poly(A) tail (4). All EV71 strains can be classified into 3 genotypes (A, B, or C) and 12 subgenotypes (A, B1 to B5, and C1 to C5) (5, 6). It is very important to understand the molecular epidemiology of EV71 in the prevention and control of EV71-mediated HFMD.

In 2017, the Changzhou Center for Disease Control and Prevention (CDC) received a report of a child with severe HFMD infection. An anal swab and a throat swab were archived and detected as positive for EV71 by real-time reverse transcription-PCR. Human EV71 (EV71/CZTN01/CHN/2017) was isolated from the throat swab, which was inoculated into rhabdomyosarcoma cell lines (RD cells). A week later, EV71-infected cells were harvested and preserved at −80°C. Viral RNA was extracted using an RNeasy minikit (Qiagen, Germany), and reverse transcription-PCR (RT-PCR) was conducted using a OneStep RT-PCR kit (Qiagen), in accordance with the manufacturer’s instructions. Six primer pairs were synthesized by the TaKaRa Biotechnology (Dalian) company. PCR products were purified with a QIAquick gel extraction kit (Qiagen), according to the manufacturer’s instructions. The purified PCR products were sequenced by the BigDye Terminator version 3.1 cycle sequencing kit (ABI) and analyzed with Sanger sequencing on an ABI 3730 DNA sequencer (ABI3730xl; Applied Biosystems, Carlsbad, CA, USA). Then, the whole-genome sequence of strain EV71/CZTN01/CHN/2017 was established by assembling overlapping fragments using the BLAST algorithm (https://www.ncbi.nlm.nih.gov/BLAST/). Nucleic acid and protein sequence alignments were analyzed using the Geneious Basic 5.6.5 software.

The complete genome sequence of strain EV71/CZTN01/CHN/2017 was composed of 7,281 nucleotides (nt), excluding the poly(A) tail. The 5′ UTR was found to be 693 nt, followed by an open reading frame (ORF) including the structural protein region P1 (2,586 nt) and the functional protein regions P2 (1,734 nt) and P3 (2,262 nt). The contents of A, U, G, and C were 26.9%, 25.1%, 23.8%, and 24.1%, respectively, and the G+C content was 48.0%. A phylogenetic tree was constructed by use of Molecular Evolutionary Genetic Analysis (MEGA) version 6.06. The results of the phylogenetic analyses suggest that strain EV71/CZTN01/CHN/2017 belongs to sub-subgenotype C4a.

### Accession number(s).

The complete genome sequence of EV71/CZTN01/CHN/2017 has been deposited in GenBank under the accession no. MG431943.
